# Supplementary material for: DMRforPairs: identifying Differentially Methylated Regions between unique samples using array based methylation profiles
Source: BMC Bioinformatics. 2014 May 15;15:141. doi: 10.1186/1471-2105-15-141 (PMC4046028; doi:10.1186/1471-2105-15-141)
Supplement: Additional file 2 — DMRforPairs output for the comparison of A431-MCF7 and NA17018-NA17105. Please start from the HTML files in each folder. Available via the BMC Bioinformatics website. [file 1471-2105-15-141-S2.zip › 1394847754114233_MOESM2_ESM/A431_MCF7/figures/24644.pdf]

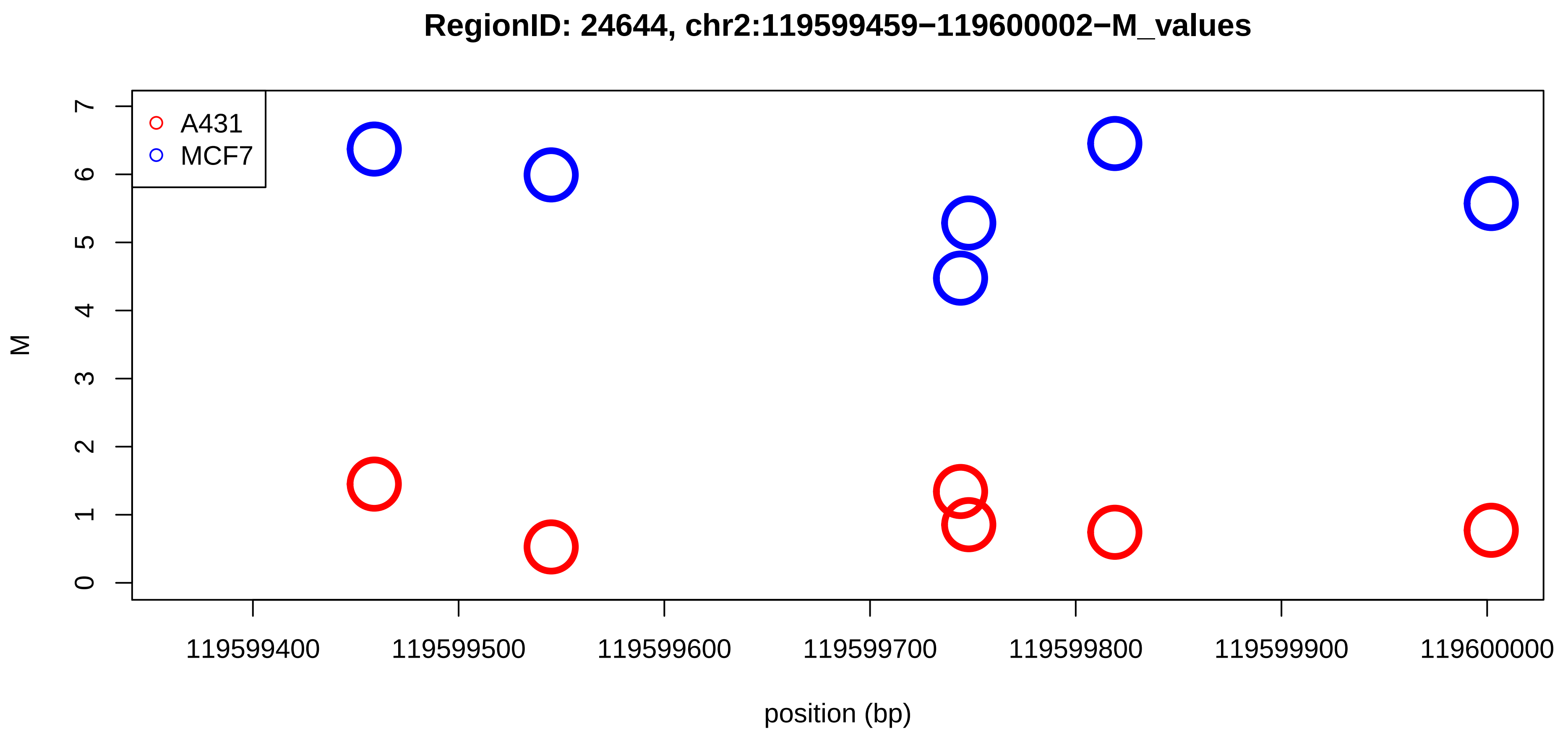
RegionID: 24644, chr2:119599459−119600002−M_values
A431
MCF7
M
0
1
2
3
4
5
6
7
119599400
119599500
119599600
119599700
119599800
119599900
119600000
position (bp)

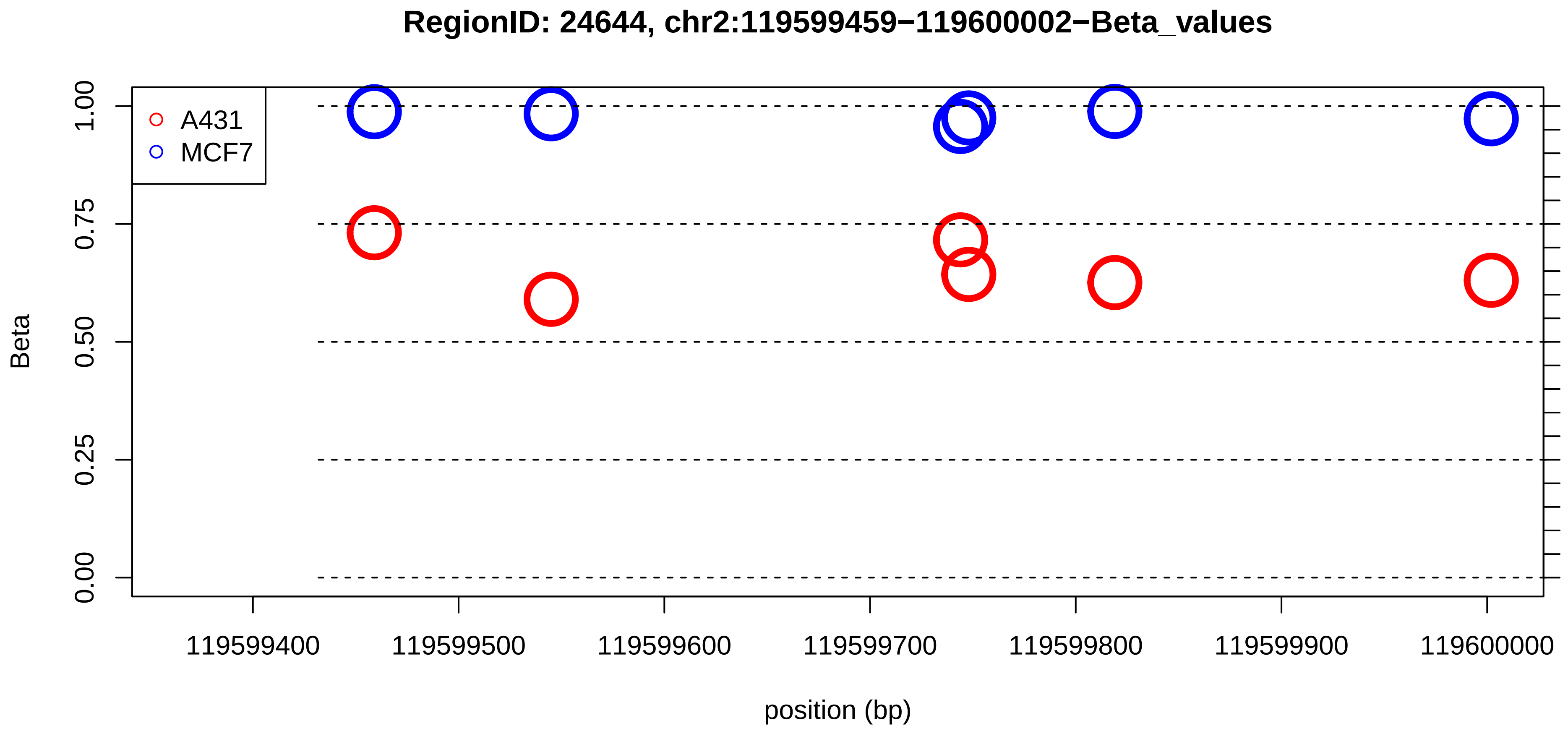
RegionID: 24644, chr2:119599459−119600002−Beta_values
A431
MCF7
Beta
0.00
0.25
0.50
0.75
1.00
119599400
119599500
119599600
119599700
119599800
119599900
119600000
position (bp)

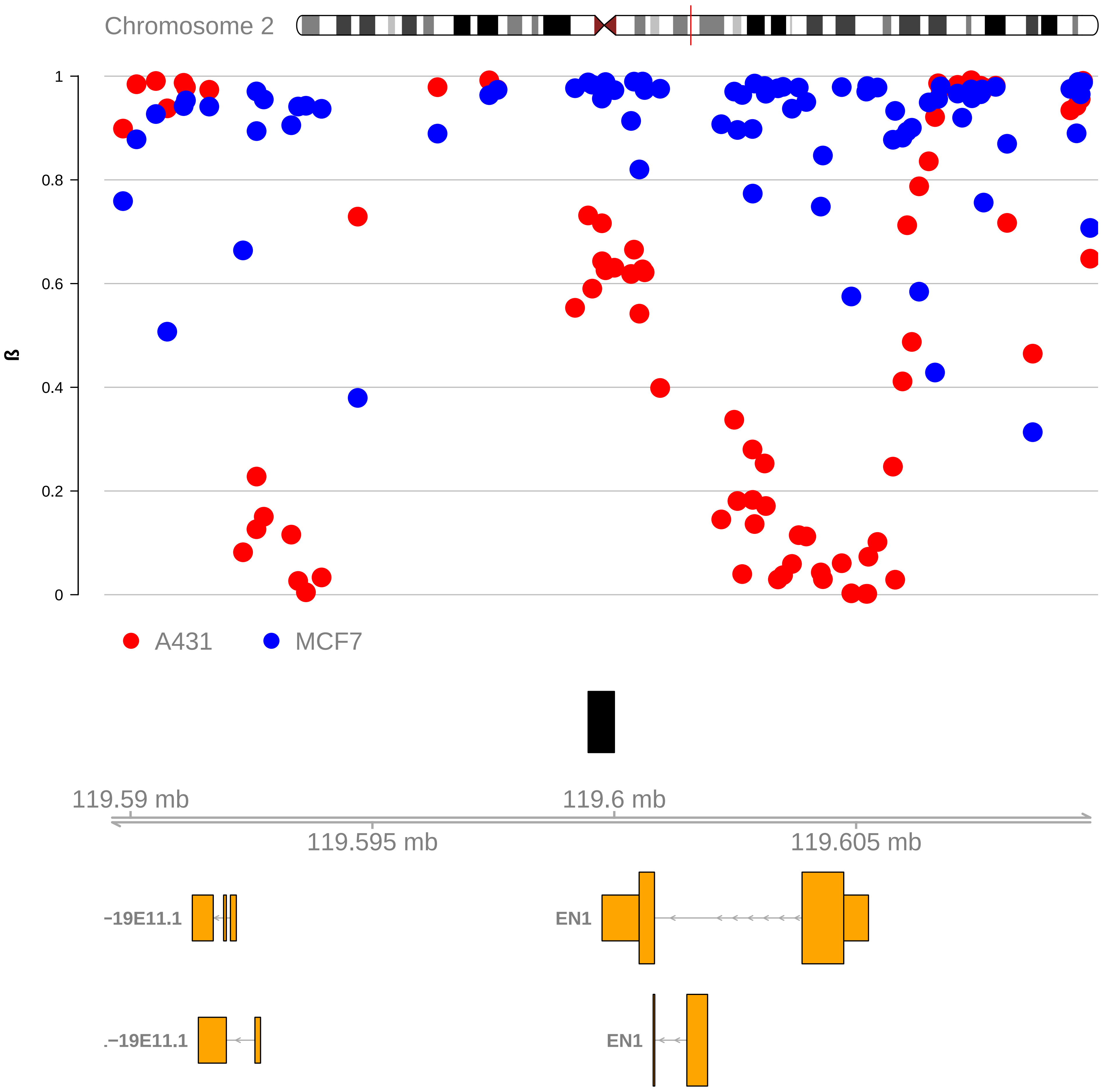

Chromosome 2
1
0.8
0.6
0.4
0.2
0
ß
A431
MCF7
119.59 mb
119.6 mb
119.595 mb
119.605 mb
−19E11.1
EN1
.−19E11.1
EN1
